# Andrographolide Ameliorates Rheumatoid Arthritis by Regulating the Apoptosis–NETosis Balance of Neutrophils

**DOI:** 10.3390/ijms20205035

**Published:** 2019-10-11

**Authors:** Xiaohong Li, Kai Yuan, Qingqing Zhu, Qingyi Lu, Haixu Jiang, Mengmeng Zhu, Guangrui Huang, Anlong Xu

**Affiliations:** 1 School of Life Sciences, Beijing University of Chinese Medicine, Beijing 100029, China; xh@bucm.edu.cn (X.L.); yuankai@bucm.edu.cn (K.Y.); qinnzh0110wai@163.com (Q.Z.); luqingyi@bucm.edu.cn (Q.L.); jianghaixu@bucm.edu.cn (H.J.); 15600790086@163.com (M.Z.); 2 State Key Laboratory of Biocontrol, Department of Biochemistry, School of Life Sciences, Sun Yat-Sen (Zhongshan) University, Guangzhou 510275, China

**Keywords:** rheumatoid arthritis, andrographolide, neutrophils, NETosis, autophagy

## Abstract

Rheumatoid arthritis (RA) is a chronic inflammatory disease characterized by symmetric polyarthritis with swelling and pain at synovial joints. In RA patients, delayed neutrophil apoptosis amplifies the inflammatory response and massively released neutrophil extracellular traps (NETs) induce tissue damage and provide self-antigens. Andrographolide (AD) is the major active labdane diterpenoid derived from *Andrographis paniculata*, which has multiple pharmacological effects, including hepatoprotection, anti-angiogenesis, anti-thrombosis, and anti-inflammation. In the present study, we investigated the effect of AD on an adjuvant-induced arthritis (AA) murine model of RA and found that AD alleviated murine arthritis by reducing neutrophil infiltration and NETosis in the ankle joints and relieved the systematic inflammation. In vitro experiments showed that AD accelerated the apoptosis of lipopolysaccharide-activated neutrophils and inhibited autophagy-dependent extracellular traps formation of neutrophils. These findings suggest that AD has considerable potential for RA therapy.

## 1. Introduction

Rheumatoid arthritis (RA) is a chronic inflammatory autoimmune disease characterized by symmetric polyarthritis with swelling and pain at synovial joints [1]. The incidence of RA is approximately 0.5–1% worldwide, with a higher incidence in women than in men [2]. Many types of immune cells have been proven to be involved in the pathogenesis of RA, including T cells, B cells, natural killer cells, neutrophils, macrophages, and dendritic cells [3]. For many years, the major contributions of neutrophils in RA were considered to be the releasing of numerous degradative enzymes and reactive oxygen species [3,4]. However, emerging studies have demonstrated that neutrophils also play critical roles in the initiation, progression, and perpetuation of RA [5].

Neutrophils granule proteins, such as myeloperoxidase (MPO) and neutrophil elastase (NE), are detected in high concentrations in RA synovial fluid, which are responsible for joint damage [6,7,8]. Synovial fluid neutrophils produce and secrete a variety of cytokines and chemokines, which are implicated in osteoclast activation and bone destruction [9]. Numerous studies have illustrated that neutrophil extracellular traps (NETs) can promote auto-immunity and aggravate tissue damage [8,9]. NETs are important sources of citrullinated autoantigens, which lead to an autoimmune response in RA [8,10]. Compared with neutrophils from healthy and osteoarthritis groups, enhanced NETosis was found in circulating and synovial fluid neutrophils from RA patients [11]. Neutrophils depletion or functional inhibition significantly ameliorates joint damage and inflammation in an arthritis murine model [12]. Therefore, neutrophils serve as an important target of RA treatment.

In the past few decades, great progress has been made in the management and treatment of RA, especially the application of biologic therapies such as tumor necrosis factor (TNF)-α-blockade agents, Janus kinase (JAK) inhibitors, and anti- interleukin (IL6) receptor antibody. However, despite the huge cost and high risk of adverse effects, only approximately 70% of RA patients get a satisfactory response to biologic therapies [13,14,15]. Thus, newer anti-arthritic therapeutic drugs are in urgent need, and natural products represent a lavish and promising source.

*Andrographis paniculata* has been used in traditional Chinese medicine for centuries. Andrographolide (AD) is the major diterpenoid bioactive compound derived from *Andrographis paniculata*, which has multiple pharmacological effects, including hepatoprotection [16], anti-angiogenesis [17], anti-thrombosis [18], and anti-inflammation [19], as well as anti-arthritis [20,21]. AD inhibits osteoclast differentiation [22], and triggers the apoptosis and cell cycle arrest of fibroblast-like synoviocytes [23]. AD also prevents oxygen radical production and the *N*-Formyl-l-methionyl-l-leucyl-l-phenylalanine (fMLP)-induced migration of neutrophils [24]. However, the underlying molecular mechanism of AD on RA remains largely unclear. In this study, we systemically investigated anti-inflammatory mechanism of AD on a murine RA model in vivo and on neutrophils in vitro.

## 2. Results

### 2.1. AD Ameliorated Adjuvant-Induced Arthritis (AA) in Mice

**Figure 1:**
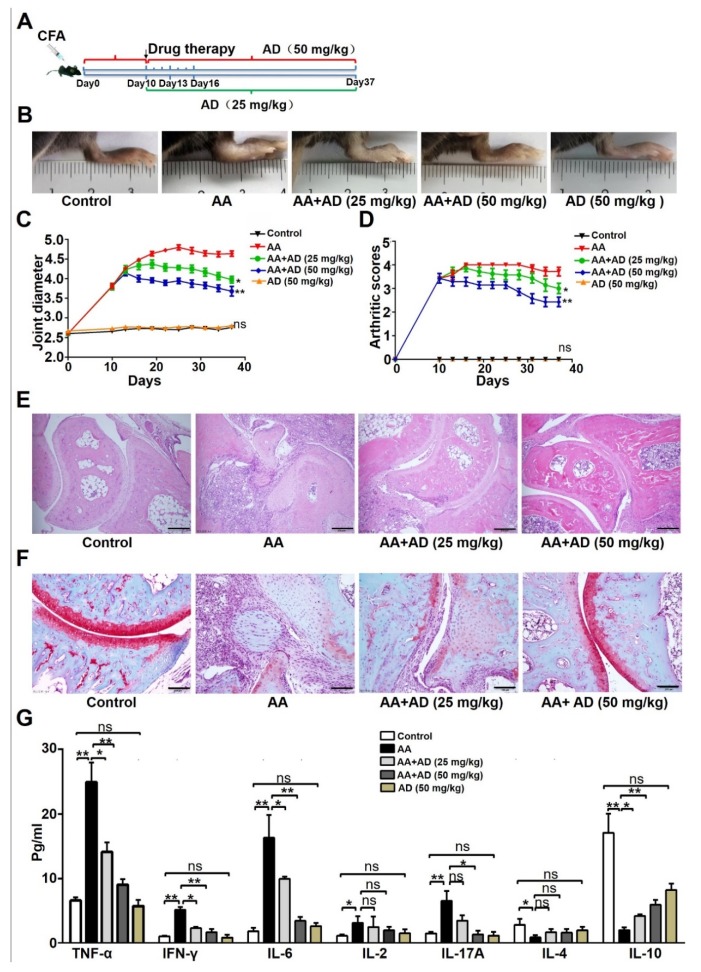
Andrographolide (AD) ameliorated adjuvant-induced arthritis in mice. (**A**) Schematic diagram of the study. (**B**) Representative pictures of the mouse hind paw on day 37. (**C**) Joint swelling was assessed by measuring the ankle joint diameter with a pocket thickness gauge (*n* = 10). Data are presented as the means ± SD. (**D**) The severity of arthritis was graded using a 0–4 arthritis scoring criterion: 0—normal, 1—slightly redness or swelling of the ankle joint, 2—moderate swelling and slightly activity limited, 3—obvious swelling and activity limited, and 4—severe swelling and activity disorder. Data are presented as the means ± SD (*n* = 10). (**E**) Hematoxylin and eosin staining of ankle joint sections of each treatment group on day 37. Images of representative sections are shown. (**F**) Assessment of articular cartilage damage by staining with Safranin O-fast green. Images of representative sections are shown. (**G**) The cytokine protein levels in the plasma of AA mice were measure using a Cytometric Bead Array (CBA) Human Chemokine Kit (*n* =10). Data represent mean ± SD (*n* = 10). * *p* < 0.05, ** *p* < 0.001. CFA: Complete Freund’s adjuvant; AA: adjuvant-induced arthritis; TNF: tumor necrosis factor, IFN: interferon; IL: interleukin.

We first investigated whether AD can ameliorate Freund’s complete adjuvant-induced arthritis (AA) (Figure 1A). As shown in Figure 1B, AD treatments significantly relieved joint edema in AA mice. The ankle joint swelling was evaluated by using the ankle joint diameter and arthritis scores every 3 days from day 10 to day 37. There was no obvious paw and joint swelling in the control group. The adjuvant induced paw edema and the ankle joint diameter increased from 2.6 mm on day 0 to 4.5 mm on day 9 post induction (Figure 1C). Consistently, the arthritis scores increased from 0 on day 0 to 4.0 on day 19 (Figure 1D), and intraperitoneal injections of AD significantly reduced the ankle joint diameter (Figure 1C) and arthritis scores (Figure 1D) compared with PBS, especially in the 50 mg/kg group (*p* < 0.05).

The ameliorating effect of AD on AA was also confirmed using HE staining and Safranin O-fast staining of the ankle joints sections. Compared with the control group, the AA group showed a large amount of leukocyte infiltration, cartilage erosion, and synovial hyperplasia in the ankle joints. AD treatment significantly alleviated those joint symptoms (Figure 1E,F). AD treatment also attenuated the up-regulation of pro-inflammatory cytokines (TNF-α, interferon (IFN)-γ, IL-6, and IL-17A) and increased the expression level of anti-inflammatory cytokine IL-10 in the serum of AA mice (Figure 1G). There was no obvious effect of AD treatment alone on mouse ankle joint diameter, arthritis score, or the expression levels of plasma cytokines (Figure 1). These results suggest that AD treatment significantly relieved the inflammation in ankle joints, as well as the whole body.

### 2.2. AD Decreases Neutrophils Infiltration

**Figure 2:**
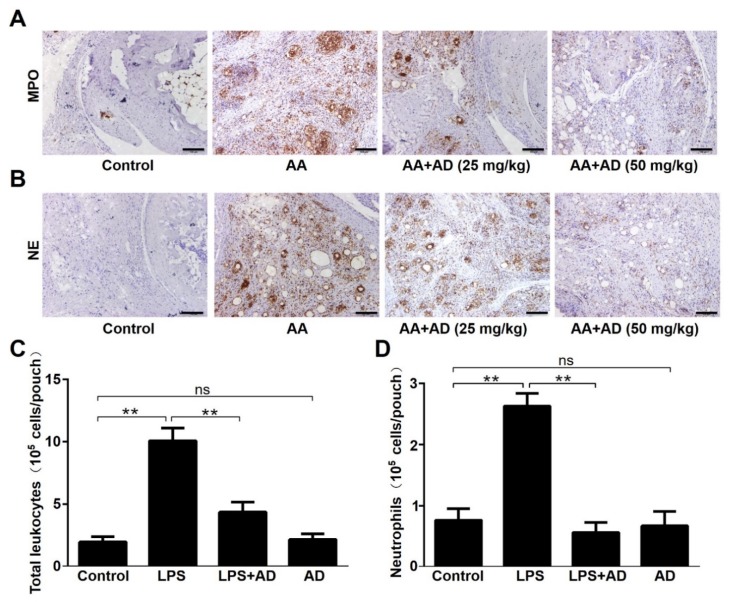
AD significantly decreased neutrophil infiltration. (**A**,**B**) Immunohistochemical analysis was performed to detect myeloperoxidase (MPO) (**A**) and neutrophil elastase (NE) (**B**) expression in the ankle joint tissue sections of each treatment group on day 37 (*n* = 10). Representative images are shown. (**C**,**D**) The air pouch assay showed that the AD (25 μM) treatment significantly suppressed lipopolysaccharide (LPS)-induced recruitment of total leucocytes (**C**) and neutrophils (**D**). The numbers of neutrophils and total leukocytes in the air pouch are expressed as means ± SD. ** *p* < 0.01 (*n* ≥ 5).

Neutrophils play pivotal roles in the pathogenesis of RA [5], and MPO and NE are well-defined markers for neutrophil infiltration. To investigate whether AD treatment influences neutrophil infiltration, immunohistochemistry was performed to evaluate the expression levels of MPO and NE in RA mice joint tissue sections. As shown in Figure 2A,B, MPO and NE expression was significantly upregulated in the RA group and AD treatment downregulated MPO and NE expression. Thus, AD decreased neutrophils infiltration in RA mouse. Air pouch assay also showed that AD treatment significantly suppressed LPS-induced recruitment of neutrophil, as well as total leucocytes (Figure 2C,D). There was no obvious effect of AD treatment alone on neutrophil recruitment in the murine air pouch model (Figure 2C,D).

### 2.3. AD Accelerates Neutrophil Apoptosis in the Presence of LPS

**Figure 3:**
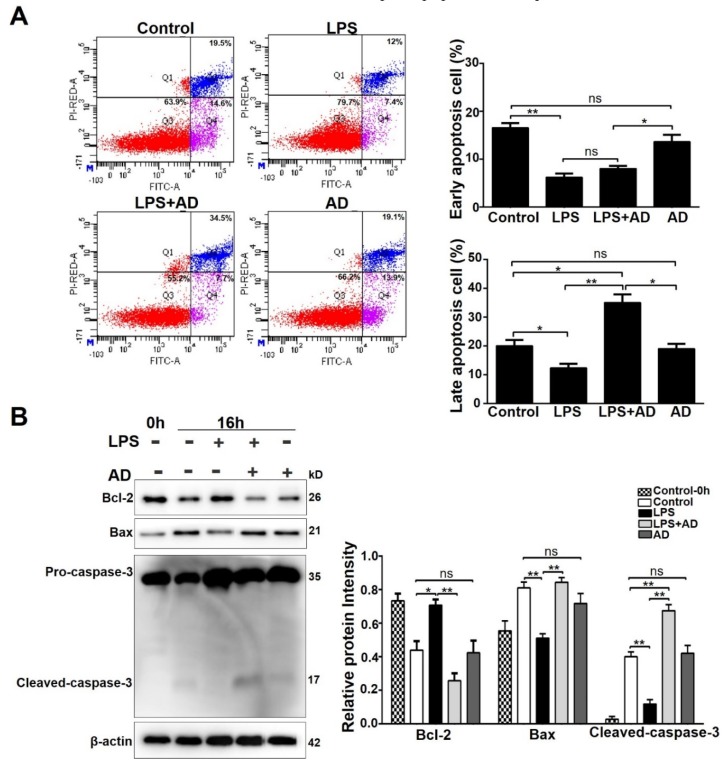
AD accelerates neutrophil apoptosis in the presence of LPS. Neutrophils were stimulated with LPS (10 ng/mL) or LPS + AD (25 μM) for 16 h, and then the cells were collected for flow cytometry or Western blot analysis. (**A**) The effects of AD on neutrophil apoptosis were analyzed using flow cytometry. (**B**) Western blot analyses of B cell lymphoma 2 (Bcl-2), Bcl-2-associated X (Bax), and caspase-3. These experiments were repeated three times and data are expressed as the mean ± SD. * *p* < 0.05, ** *p* < 0.01.

Neutrophils are short-life leukocytes that undergo spontaneous apoptosis in the peripheral blood [25]. However, when they migrate into RA joints, the apoptosis of neutrophils is delayed, which prolongs the inflammation and increases the release of tissue-damage molecules, such as reactive oxygen species, elastase, and metalloproteases [26,27]. To investigate whether AD can reverse the neutrophil apoptosis delay, purified neutrophils were incubated with LPS or LPS + AD for 16 h, then were stained with Annexin V/PI and analyzed using flow cytometry. As shown in Figure 3A, the LPS treatment significantly reduced the early apoptosis (annexin V^+^, PI^−^) and late apoptosis (annexin V^+^, PI^+^) of neutrophils. LPS + AD treatment significantly increased the late apoptosis of neutrophils and the number of late apoptosis cells was even more than that of the control group. Western blot analyses showed that compared with the LPS treatment, the LPS + AD treatment significantly reduced anti-apoptotic protein Bcl-2 expression and enhanced pro-apoptotic protein Bax expression, which lead to the cleavage and activation of caspase-3. The expression level of cleaved-caspase-3 in the LPS + AD treatment group was even higher than that of the control group (Figure 3B). There was no obvious effect of AD treatment alone on the apoptosis of neutrophils compared with the control group (Figure 3). These results showed that AD accelerated neutrophil apoptosis in the presence of LPS.

### 2.4. AD Inhibited PMA-Induced NETosis

**Figure 4:**
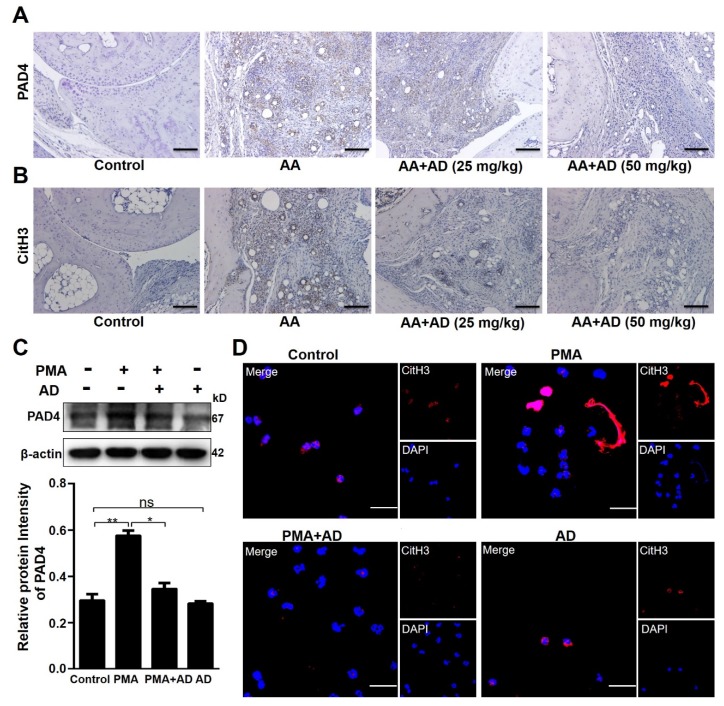
AD inhibited peptidylarginine deiminase 4 (PAD4) expression and histone 3 citrullination. (**A**,**B**) Immunohistochemical analysis was performed to detect PAD4 (**A**) and citrullinated histone 3 (CitH3) (**B**) expression in the ankle joint tissue sections of each treatment group on day 37 (*n* = 10). Representative images are shown. (**C**) PAD4 expression levels were measured using Western blotting and the relative gray values were quantified using Image J. The β-actin was an internal control. Data indicate mean ± SD of three independent experiments, * *p* < 0.05, ** *p* < 0.01. (**D**) Phorbol 12-myristate 13-acetate (PMA)-induced neutrophil extracellular trap (NET) formation was visualized via staining neutrophils with 4’,6-diamidino-2-phenylindole (DAPI) (blue) and an anti-CitH3 antibody (red) and observed using confocal microscopy. Scale bar represents 10 μm. Representative images obtained from more than three experiments are shown.

NETs are extracellular web-like DNA decorated with MPO, NE, and other antimicrobial proteins. NETs are released from neutrophils during inflammation via a distinct process termed NETosis [7,28,29]. Besides granule enzymes, NETs also externalize various autoantigens, such as citrullinated histone 3 (CitH3), which induces specific autoantibodies and autoimmune responses in RA patients [30,31,32]. Peptidylarginine deiminase 4 (PAD4) is a nuclear citrullinating enzyme, which transforms protein l-arginine to l-citrulline and plays an important role in RA pathogenesis [33,34]. PAD4 is also a target of autoantibodies in a subgroup of RA patients and anti-PAD4 autoantibodies can serve as a severity biomarker of RA [35]. Immunostaining of RA mouse ankle joint tissue sections showed that PAD4 was up-regulated in RA mice and the AD treatment significantly reduced PAD4 expression (Figure 4A). Consistently, AD also suppressed CitH3 expression (Figure 4B). Then, purified neutrophils were used to test whether AD could directly suppress the expression of PAD4 and CitH3 in neutrophils. Western blot analyses showed that AD suppressed PMA-induced up-regulation of PAD4 (Figure 4C). Immunostaining analyses of CitH3 showed that AD treatment reversed PMA-induced histone 3 citrullination (Figure 4D).

**Figure 5:**
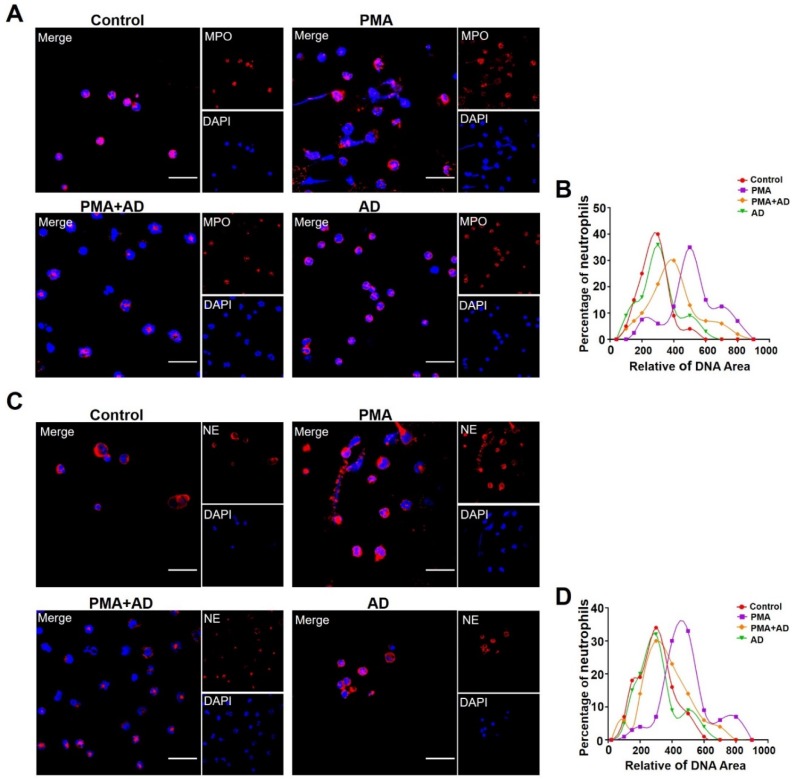
(**A**,**C**) PMA-induced NET formation was visualized via staining neutrophils with DAPI (blue) and an anti-MPO antibody ((**A**); red) or anti-NE antibody ((**C**); red) and observed using confocal microscopy, Scale bar represents 10 μm. Representative images obtained from more than three experiments are shown. (**B**,**D**) Relative DNA area of 100 neutrophils in A (**B**) and C (**D**). The data were obtained from three independent experiments.

Consistently, immunostaining analyses of MPO and NE showed that AD treatment reduced the PMA-induced NETosis of neutrophils (Figure 5A,C). Quantification of NETosis with relative DNA area showed that AD treatment significantly suppressed the PMA-induced nuclear de-condensation of neutrophils (Figure 5B,D). There was no obvious effect of AD treatment alone on the levels of PAD4, CitH3, and NETosis in neutrophils compared with the PBS treatment (Figure 4 and Figure 5).

### 2.5. AD Inhibited Neutrophil Autophagy

**Figure 6:**
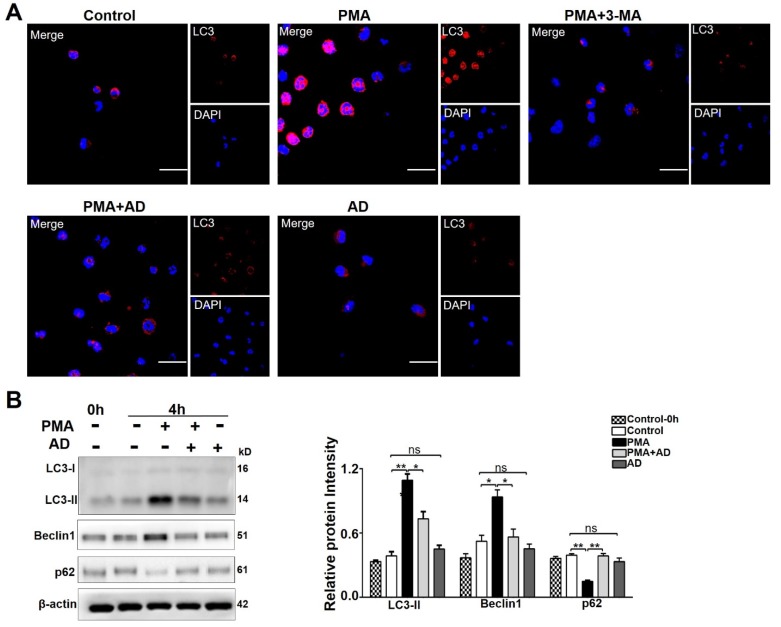
AD inhibited neutrophil autophagy. (**A**) Immunostaining of purified neutrophils with anti- microtubule-associated protein 1A/1B-light chain 3 (LC3) antibody. Representative images of more than three experiments are shown. Scale bar represents 10 μm. (**B**) Western blot analyses of LC3, Beclin1, and p62 in neutrophils treated with PMA (25 nM) or PMA (25 nM) + AD (25 μM) for 4 h. The relative gray values were quantified using Image J. The β-actin was an internal control. Data indicate mean ± SD of three independent experiments, * *p* < 0.05, ** *p* < 0.01. 3-MA: 3-methyladenine.

Autophagy is a vital cellular degradation mechanism responsible for the metabolism of the cells themselves and the renewal of certain organelles [36]. Previous studies have demonstrated that autophagy is required for NETs formation [37,38,39]. RA patients had significantly higher levels of autophagy in circulating leukocytes compared with healthy controls [40,41]. Immunostaining of purified neutrophils with anti-microtubule-associated protein 1A/1B-light chain 3 (LC3) antibody showed that AD treatment significantly reduced PMA–induced up-regulation of LC3 (Figure 6A,B). Western blot analyses showed that AD treatment down-regulated the expression levels of LC3-II and Beclin1, and up-regulated the expression level of p62 (Figure 6C). These results suggested that AD could significantly inhibit the autophagy of neutrophils.

## 3. Discussion

AD is a major bioactive constituent extracted from *Andrographis paniculata*, which belongs to the family of *Acanthaceae*. Despite recent progress in understanding the anti-arthritic effects of AD, the immunological mechanism that regulated neutrophils activities was unknown. In this study, we comprehensively investigated the role of AD on neutrophils in adjuvant-induced murine arthritis (AA) models. AD-treated AA mice exhibited a remarkable reduction in clinical arthritic scores, neutrophil infiltration, and bone destruction.

Neutrophils play important roles in the onset and perpetuation of RA [42]. The cytokines and mediators produced by neutrophils could amplify the inflammatory response in RA [5]. Previous studies demonstrated that inflammatory mediators, including cytokines, chemokines, and reactive oxygen species (ROS), have pleiotropic effects on inflammation progress [43,44]. TNF-α, IL-6, IL-17A, and IFN-γ have important effects in recruiting and activating neutrophils. These inflammatory cytokines could amplify the immune response by inducing the production of other cytokines and chemokines [45,46,47]. These cytokines are also involved in the pathogenesis of RA by activating chondrocytes and osteoclasts [48,49]. The IFN-γ expression level is significantly higher in RA patients after disease onset compared with health control [50]. As a factor of anti-inflammatory cytokines, IL-10 has the capacity to inhibit neutrophils activation, migration, and degranulation [51,52]. Targeting the inflammatory cytokines including TNF-α, IL-17A, and IL-6 are a pivotal strategy for RA treatment. TNF-α inhibitors (Adalimumab, Etanercept, Certolizumab) and IL-6 inhibitors (Tocilizumab) have been licensed by the Food and Drug Administration (FDA) for treating RA patients [53]. In this study, we found that AD treatment decreased the levels of pro-inflammatory cytokines (TNF-α, IFN-γ, IL-6, IL-17A), while it increased the level of anti-inflammatory cytokine IL-10 in the plasm of AA mice. These data indicated that AD had systematic anti-inflammatory effects in AA mice.

Furthermore, we investigated the mechanism of AD in the AA murine model. First, we discovered that AD inhibited the migration of neutrophils. The steps of neutrophils recruitment cascade include tethering, rolling, adhesion, crawling, and transmigration [54]. Neutrophil migration is an important stage in the inflammatory process of RA [27]. In the circulating blood cells, neutrophils are the first ones to infiltrate into the joints. They are the most plentiful cells in the synovial fluid. Several molecules, such as E-selectin, P-selectin, and IL-6, are involved in the massive migration of neutrophils into the joints. MPO and NE are regarded as markers for neutrophils infiltration in animal model. In the present study, the expression levels of MPO and NE were increased in the RA group compared with the control group. AD treatment significantly reduced MPO and NE expression compared with the RA group. The results indicated that AD could reduce neutrophil infiltration to the RA joints. We also found that AD inhibited LPS-induced recruitment of neutrophils in the air pouch assay. Taken together, AD could reduce neutrophils recruitment in RA.

Second, we demonstrated that AD accelerated neutrophil apoptosis in the presence of LPS. Naive neutrophils have a very short lifespan of 6 to 18 h before undergoing constitutive apoptosis [54]. However, in the synovial fluid of RA joints, the apoptosis of neutrophils are delayed [4]. Activated synovial fluid neutrophils could increase the inflammatory status and promote joint destruction. Neutrophils in the synovial fluid exhibited a higher expression of anti-apoptotic proteins (Bcl-2) and a lower expression of pro-apoptotic proteins (Bax, cleaved caspase-3) [27]. In our study, we showed that AD could reduce the expression of anti-apoptotic protein Bcl-2. Bcl-2 could be phosphorylated by activated extracellular signal-regulated kinase (ERK)1/2 on Serine 70, which blocks the ubiquitin-dependent proteasomal degradation of Bcl-2 [55]. AD inhibits the activation of ERK1/2 [20], which reduces the phosphorylation of Bcl-2 and promotes the degradation of Bcl-2. Additionally, AD increased pro-apoptotic protein Bax and cleaved caspase-3 expression. It is of note that the percentage of late apoptosis neutrophils and the expression level of cleaved-caspase-3 in the LPS + AD treatment group was even higher than the control group, which demonstrated the AD treatment accelerated neutrophil apoptosis in the presence of LPS.

Third, we explored the effects of AD in inhibiting PMA-induced NETosis. NETosis is a featured form of death of neutrophils [30]. NETs are extracellular fibrous DNA networks combined with granular and nuclear proteins. In recent years, researchers have proven that NETs play important roles in the pathogenesis of RA [56]. NETs are correlated with the production of anti-citrullinated protein antibodies (ACPAs) in RA [32]. NETs are an important source of autoantigens to stimulate ACPA production. ACPAs also facilitate the release of peptidyl arginine deiminases (PADs) of neutrophils, which transforms the protein L-arginine to L-citrulline and forms a vicious circle [33]. Apart from autoantibody production, NETs also induce inflammatory cytokine production to enlarge inflammatory response [56]. Recently, researchers reported that NETosis-derived products could be used to assess therapeutic effectiveness in RA patients [57]. MPO, NE, and citrullinated histone H3 (CitH3) are major component of NETs, which are considered biomarkers of NETosis [30]. In this study, we proved that AD significantly inhibited NETs formation and NETs-associated MPO, NE, and CitH3 release. PAD4 is an important enzyme that catalyzes protein citrullination. PAD4 is essential for the formation of NETs. PAD4-mediated histone citrullination is considered to promote NETs formation by facilitating chromosomal DNA expulsion and chromatin decondensation. Many studies have identified that PAD4 was a potential therapeutic target to treat RA [57]. PAD4 expression level is positively correlated with the severity of RA [58]. In our study, we found that AD significantly downregulated the expression of PAD4 in the ankles of RA mice.

Autophagy is a well-known intracellular mechanism for degradation and energy recycling [36]. It is an important regulatory mechanism in immune responses [38]. Neutrophil autophagy facilitates the formation of NETs [37]. LC3-II is the most important parameter to test autophagic flux, which is associated with the maturation of autophagosome [36]. Beclin1 regulates the autophagy and membrane trafficking involved in several pathological and physiological processes [59]. P62 is considered an autophagy substrate used as a reporter of autophagy activity [60]. In this study, we proved that AD could significantly inhibit the autophagy of neutrophils. We found that AD treatment down-regulated the expression levels of LC3-II and Beclin1, and up-regulated the expression of p62. AD treatment significantly reduced the phosphorylation of p38 mitogen-activated protein kinase (MAPK) and ERK1/2 [20], which is required for the activation of reduced nicotinamide adenine dinucleotide phosphate (NADPH) oxidase and ROS production [61], thus reducing the NET formation in a ROS-dependent manner. Autophagy is also responsible for the degradation of Bax protein under basal conditions [62], and the inhibition of neutrophil autophagy maybe the reason for the up-regulation of Bax after AD treatment.

## 4. Materials and Methods

### 4.1. Reagents and Antibodies

Complete Freund’s adjuvant (CFA), lipopolysaccharide (LPS), andrographolide, and phorbol 12-myristate 13-acetate (PMA) were purchased from Sigma-Aldrich (St Louis, MO, USA). 3-methyladenine (3-MA) was purchased from Selleck Chemicals LLC (Houston, TX, USA). Mouse Th1/Th2/Th17 cytometric bead array (CBA) kits were purchased from BD Biosciences (Franklin Lakes, NJ, USA). Anti-myeloperoxidase, anti-neutrophil elastase, anti-LC3, anti-Beclin1, anti-p62, anti- Bcl-2, anti-Bax, anti-rabbit-horseradish peroxidase, and immunoglobulin G antibodies were purchased from Abcam (Cambridge, MA, USA), and anti-cleaved caspase-3 was purchased from Cell Signaling Technology (Danvers, MA, USA).

### 4.2. Animals

C57BL/6 mice (female, 8 weeks old) were purchased from the Chinese Academy of Military Medical Sciences (Beijing, China). Mice were maintained in standard housing cages under specific pathogen-free conditions. All experimental procedures were reviewed and approved by the Animal Care and Use Committee of the Beijing University of Chinese Medicine (ethical approval number: BUCM-4-2018060416-2020, approved date: 4 June, 2018). All experiments were performed in accordance with the institutional guidelines for the Care and Use of Laboratory Animals.

### 4.3. Induction of Adjuvant-Induced Arthritis (AA)

Twenty microliters of CFA was injected into the joint space and four periarticular sites, respectively. Forty mice were randomly divided into four groups: control group (PBS), model group (AA), 25 mg/kg AD group (AD (25 mg/kg)), and 50 mg/kg AD group (AD (50 mg/kg)). To assess the ankle joint swelling, the joint diameters were measured with a pocket thickness gauge (Mitutoyo, Kawasaki, Japan). Arthritis severity was assessed using arthritis using a 0–4 scoring criterion scale: 0—normal, 1—slightly redness or swelling of the ankle joint, 2—moderate swelling and slightly activity limited, 3—obvious swelling and activity limited, and 4—severe swelling and activity disorder.

### 4.4. Histochemistry and Immunohistochemistry

Mice were sacrificed on day 37. The ankle joints were collected, fixed in 4% paraformaldehyde for 48 h, decalcified in 10% ethylenediaminetetraacetic acid solution, and embedded in paraffin. The tissue sections were stained with hematoxylin and eosin (HE), Weigert’s Iron Hematoxylin solution (Sigma-Aldrich) and Fast Green solution (Sigma-Aldrich) for histopathological analysis. The MPO and NE expression levels were examined using immunohistochemistry with anti-NE and anti-MPO antibodies in accordance with the instructions of the manufacturer.

### 4.5. Cytokine Analysis

Serum samples were obtained from mice’s blood in each group on day 37. The concentrations of cytokines (TNF-α, IFN-γ, IL-6, IL-2, IL-17A, IL-4, and IL-10) were measured with the Mouse Th1/Th2/Th17 Cytometric Bead Array (CBA) Human Chemokine Kit following the manufacturer’s introduction.

### 4.6. Air Pouch Experiments

C57BL/6 male mice (≥5/group) were anaesthetized using chloral hydrate, and 3 mL of sterilized air (filtered through a 0.22-μm filter; Millipore, Billerica, MA, USA) was injected into the skin on days 0 and 3 with a 26-gauge needle to produce an air pouch on the back. On day 6, 1 mL of buffer (control) or 1 μg/mL LPS with or without 25 μM AD was injected into the air pouches of mice 6 h before the mice were euthanized via CO_2_ asphyxiation. The air pouches were washed once with 1 mL and then twice with 2 mL of Hanks’s balanced salt solution (HBSS) containing 10 mM ethylenediamine tetraacetic acid (EDTA), and the exudates were centrifuged at 100× *g* for 10 min at room temperature. Cells were resuspended in 1 mL of HBSS containing 10 mM EDTA and stained with Wright’s stain to quantify the neutrophil populations.

### 4.7. Isolation of Mouse Neutrophils and Culture

Peritoneal exudate cells were collected from lavage fluid of mice inoculated intraperitoneally with 1 mL of 10% protease peptone 10–12 h previously. Cells were resuspended in 1 mL of RPMI-1640 supplemented with 10% fetal bovine serum (FBS), layered onto a two-step (54.8%/70.2%) discontinuous Percoll gradient, and centrifuged at 1500× *g* for 30 min at 22 °C. Neutrophils (≥95%, approximately 1 × 10^7^ neutrophils/mouse) were recovered from the lower interface and were cultured at 37 °C with 5% CO_2_ in humidified incubator.

### 4.8. Western Blot Analysis

Neutrophils were collected and were lysed with radioimmunoprecipitation assay (RIPA) buffer containing protease inhibitor. Protein concentration was measured using a BCA Protein Assay Kit (Thermo Fisher Scientific, Tewksbury, MA, USA). Samples were separated using 10% sodium dodecyl sulfate polyacrylamide gel electrophoresis (SDS-PAGE) and were transferred to nitrocellulose membranes (Millipore, Billerica, MA, USA). The membranes were blocked with 5% non-fat dried milk for 1 h at room temperature and were incubated with primary antibodies against Bax, Bcl-2, cleaved-caspase-3, Beclin1, p62, LC3, or β-actin at 4 °C overnight. After three washes, the membranes were incubated with horseradish peroxidase (HRP)-conjugated secondary antibodies for 1 h at room temperature. The proteins were detected using an enhanced chemiluminescence reagent (GE Healthcare, Chicago, IL, USA). The intensities of protein bands were quantified using ImageJ software (version 1.43 National Institutes of Health, Bethesda, MD, USA).

### 4.9. Immunofluorescence Assay

Neutrophils were seeded at a density of 1 × 10^6^ cells/mL on glass coverslips in a six-well plate. The cells were incubated with or without AD (25 μM) and PMA (25 nM) for 4 h. Then, cells were fixed with 4% paraformaldehyde for 15 min and were permeabilized with 0.1% Triton X-100 in PBS for 10 min. After blocking with 5% BSA/PBS for 1 h, cells were incubated with anti-MPO, anti-NE, or anti-LC3 antibodies at 4 °C overnight and subsequently with Alexa-FluorVR-555-labeled secondary antibody (Invitrogen, Carlsbad, CA, USA) at room temperature for 1 h. Nuclei were counterstained with 4’,6-diamidino-2-phenylindole (DAPI) for 5 min and images were captured using a confocal microscope (FV1000, Olympus, Japan). Relative DNA area was measured with Image-Pro Plus software (version 6.0, Media Cybernetics Corporation, Rockville, MD, USA).

### 4.10. Flow Cytometry Analysis

Purified neutrophils were resuspended in culture medium at a density of 10^6^ cells and 2 mL of cell suspension was plated in a six-well plate per well. Cells were treated with LPS and/or AD for 16 h. Cells were resuspended in 200 μL of 1× binding buffer after being washed twice with cold PBS. Fluorescein isothiocyanate (FITC)-annexin V and propidium iodide (PI) (5 μL each) were added and incubated for 15 min at room temperature (RT) (25 °C) in the dark with a gentle vortex. Then, cells were washed and analyzed using flow cytometry within 1 h. annexin V^+^ and PI^−^ cells were considered as early apoptosis cells and annexin V^+^ and PI^+^ cells were considered as late apoptosis cells.

### 4.11. Statistical Analysis

Data were expressed as the mean ± standard deviation (SD). Inter group comparisons were performed by using one-way analysis of variance. If the data did not satisfy a normal distribution, the rank sum test was used. A *p*-value less than 0.05 was considered to be significant.

## 5. Conclusions

**Figure 7:**
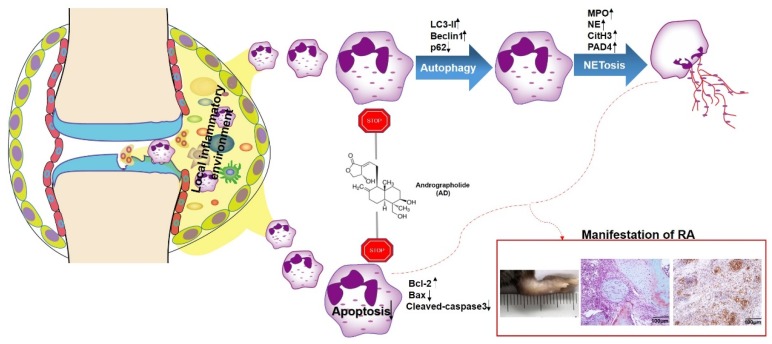
A graphical summary shows how andrographolide ameliorates rheumatoid arthritis by regulating the apoptosis–NETosis balance of neutrophils.

The present study investigated the anti-arthritis mechanism of AD in a murine RA model and our results showed that AD alleviated murine arthritis by promoting neutrophil apoptosis and suppressed the autophagy-dependent NETosis of neutrophils (Figure 7). These findings suggest that AD has considerable pharmaceutical potential for RA treatment.
